# Mechanistic Modeling of Dose and Dose Rate Dependences of Radiation-Induced DNA Double Strand Break Rejoining Kinetics in *Saccharomyces cerevisiae*

**DOI:** 10.1371/journal.pone.0146407

**Published:** 2016-01-07

**Authors:** Igor Shuryak

**Affiliations:** Center for Radiological Research, Columbia University, New York, NY, United States of America; Institut Pasteur, FRANCE

## Abstract

Mechanistic modeling of DNA double strand break (DSB) rejoining is important for quantifying and medically exploiting radiation-induced cytotoxicity (e.g. in cancer radiotherapy). Most radiation-induced DSBs are quickly-rejoinable and are rejoined within the first 1–2 hours after irradiation. Others are slowly-rejoinable (persist for several hours), and yet others are essentially unrejoinable (persist for >24 hours). The dependences of DSB rejoining kinetics on radiation dose and dose rate remain incompletely understood. We hypothesize that the fraction of slowly-rejoinable and/or unrejoinable DSBs increases with increasing dose/dose rate. This radiation-dependent (RD) model was implemented using differential equations for three DSB classes: quickly-rejoinable, slowly-rejoinable and unrejoinable. Radiation converts quickly-rejoinable to slowly-rejoinable, and slowly-rejoinable to unrejoinable DSBs. We used large published data sets on DSB rejoining in yeast exposed to sparsely-ionizing (electrons and γ-rays, single or split-doses, high or low dose rates) and densely-ionizing (α-particles) radiation to compare the performances of the proposed RD formalism and the established two-lesion kinetic (TLK) model. These yeast DSB rejoining data were measured within the radiation dose range relevant for clonogenic cell survival, whereas in mammalian cells DSB rejoining is usually measured only at supra-lethal doses for technical reasons. The RD model described both sparsely-ionizing and densely-ionizing radiation data much better than the TLK model: by 217 and 14 sample-size-adjusted Akaike information criterion units, respectively. This occurred because: the RD (but not the TLK) model reproduced the observed upwardly-curving dose responses for slowly-rejoinable/unrejoinable DSBs at long times after irradiation; the RD model adequately described DSB yields at both high and low dose rates using one parameter set, whereas the TLK model overestimated low dose rate data. These results support the hypothesis that DSB rejoining is progressively impeded at increasing radiation doses/dose rates.

## Introduction

Mechanistic quantitative modeling of DNA double strand break (DSB) rejoining kinetics is important for predicting radiation-induced cytotoxicity and for exploiting it (e.g. in cancer radiotherapy) [1–5], as well as for assessment of radiation risks at low doses [6–8]. Accumulating evidence suggests that DSB rejoining occurs via multiple biochemical pathways, often with multiphasic kinetics [9–12]. Some DSBs may be more difficult to rejoin than others due to their “complexity”, which can be “chemical” (e.g. radiation-induced damage to DNA bases and/or chromatin near the DSB) and/or “spatial” (e.g. location of the DSB in heterochromatin vs. euchromatin, presence of multiple DSBs within one chromatin loop, short length of DNA fragment between two DSBs) [13–22]. The dependence of DSB complexity on radiation quality (e.g. linear energy transfer) has received sustained attention [15, 23–27]. In contrast, the dependences of DSB rejoining kinetics on radiation dose and dose rate remain incompletely understood [13, 28, 29].

Mechanistic quantitative analysis of DSB rejoining (and clonogenic cell survival) is often performed using kinetic models which describe the rates of change of the average number of DSBs per cell during and/or after radiation exposure. Many such models have been proposed, some of which attempt very detailed descriptions of molecular machinery involved in DSB repair [5, 30–33]. Simpler formalisms, such as the two-lesion kinetic (TLK) model [34, 35], generalize earlier repair-misrepair (RMR) [36] and lethal-potentially-lethal (LPL) [37] models to multiple DSB classes. The spectrum of DSB complexity is modeled by these classes, each of which is allowed to have its own rates of induction and removal. Such models aim to capture the main rate-limiting steps in DSB rejoining in a sufficiently parsimonious manner to be easily applicable for quantitative analysis of experimental data sets, which are often quite limited in the range of radiation doses and/or dose rates.

Here, we hypothesize that the understanding of how DSB rejoining depends on radiation dose and dose rate can be enhanced by incorporating into kinetic models a new mechanism, whereby the fraction of slowly-rejoinable and/or unrejoinable DSBs increases with increasing dose and/or dose rate. The hypothetical mechanism can occur, for example, due to a gradually increased fraction of spatial DSB clustering along chromosomes [28]. Such clustering, as implemented in the The Giant LOop Binary LEsion (GLOBLE) model [17, 28], can lead to higher DNA damage complexity, which in turn is then connected with slower rejoining. In addition, dose-dependent accumulation of radiation damage to chromatin and/or to the enzymatic repair complexes themselves can also occur [24, 27].

We mathematically implemented this radiation-dependent (RD) model, and compared its performance to that of the TLK model using large published data sets on DSB rejoining in yeast (*Saccharomyces cerevisiae*) exposed to sparsely-ionizing (30 MeV electrons, single or split-doses, high dose rate; γ-rays, low dose rate) [38, 39] and densely-ionizing (α-particles) radiation [40, 41]. The results (presented below) show that the RD model is able to describe these data dramatically better than the TLK model, supporting the hypothesis that the fraction of slowly-rejoinable and/or unrejoinable radiation-induced DSBs depends on radiation dose/dose rate. This conclusion, which has potential clinical relevance for modeling and optimizing cancer radiotherapy, should be tested further in mammalian cells, which have somewhat different DSB repair machinery than yeast.

## Materials and Methods

### Data sets

Large data sets on radiation-induced DSB rejoining kinetics, preferably encompassing a wide range of radiation types, doses, dose rates, and rejoining times, are needed to generate statistically robust comparisons of kinetic models. DSB rejoining has been measured by several methods: e.g. neutral filter elution, pulsed-field gel electrophoresis, or by surrogate markers such as *γ* H2AX foci [22, 29, 42–44]. In mammalian cells, the first two methods produce reliable results only at supra-lethal radiation doses (generally > 20 Gy), at which cells remain metabolically functional for some time, but are clonogenically dead [22, 29, 31]. The third method is applicable to lower doses, but the kinetics of foci accumulation and decay can be quite different from the underlying DSB rejoining kinetics. In *Saccharomyces cerevisiae*, which has a much smaller genome than mammalian cells, DSB rejoining can be quantified directly at doses relevant for clonogenic cell survival [45].

Extensive data sets on DSB rejoining in *S*. *cerevisiae* were produced by Frankenberg-Schwager et al. [38–41]. Petite mutant yeast (diploid strain 211*B) lack mitochondrial DNA, which facilitates radio-labeling of nuclear DNA. The yeast cells were lysed on top of a neutral sucrose gradient (5–20%) and the released DNA was sedimented by centrifugation [38]. Gradients were fractionated onto glass fiber filters, dried, and treated with a toluene-based scintillation liquid. The percentage of total radioactivity of DNA in each fraction as a function of the sedimented volume yielded DNA profiles which were used to determine the number of radiation-induced DSBs. The average number of DSBs per molecular mass of DNA was calculated by computer simulation of random breakage as applied to the DNA of unirradiated cells and by fitting these calculated curves to the DNA profiles obtained from irradiated cells [38].

**Fig 1:**
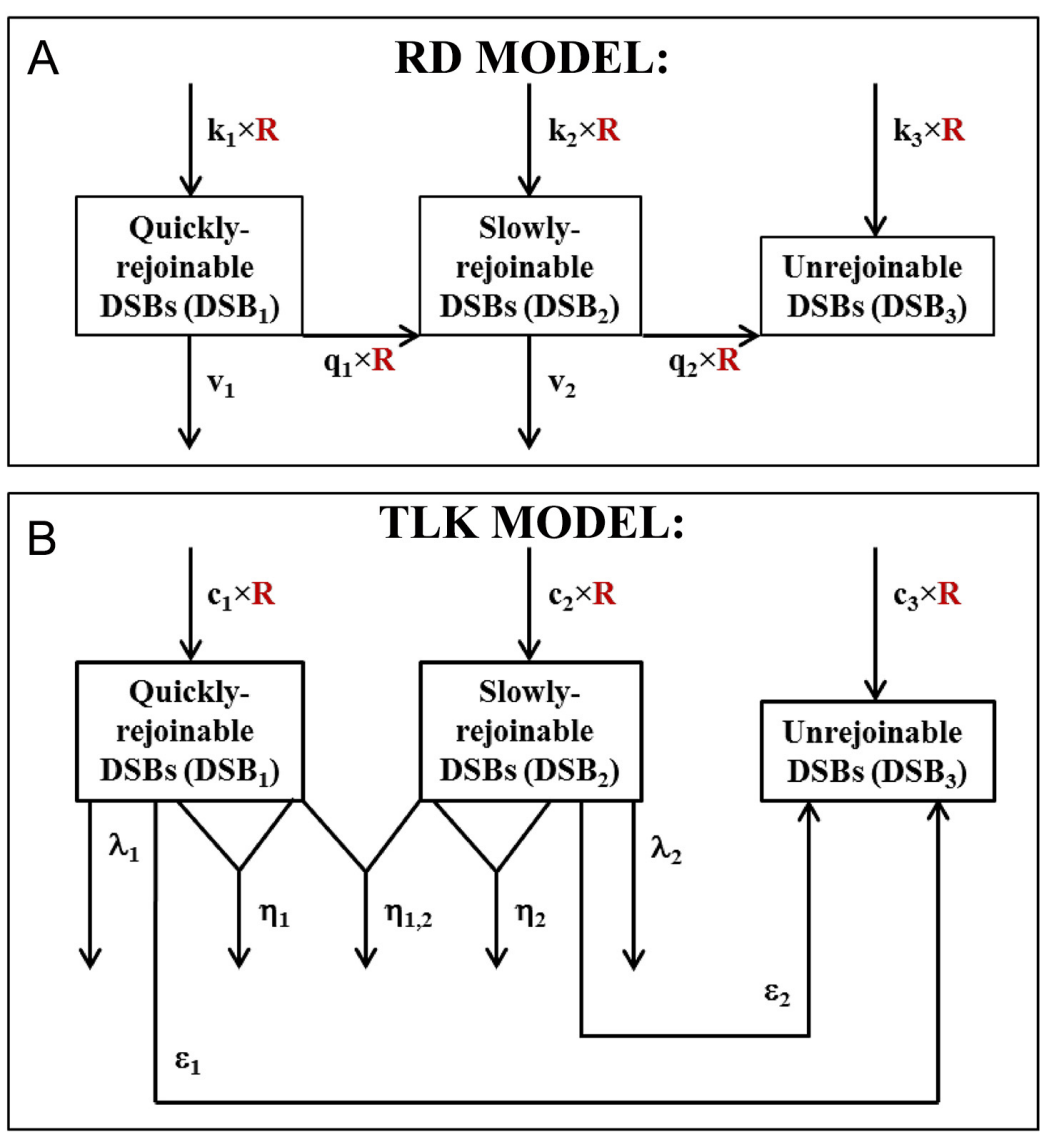
Schematic representations of the RD (panel A) and TLK (panel B) models. In the RD model, radiation (with dose rate R) produces three DSB classes (DSB_1_, DSB_2_, DSB_3_) with yields (per unit dose) of *k*_1_, *k*_2_ and *k*_3_, respectively. DSB_1_ and DSB_2_are rejoined with rates *v*_1_ and *v*_2_, respectively; DSB_3_ are unrejoinable. Radiation converts DSB_1_ to DSB_2_ and DSB_2_ to DSB_3_ with rates proportional to parameters *q*_1_ and *q*_2_, respectively. In the TLK model, radiation also produces three DSB classes with yields (per unit dose) of *c*_1_, *c*_2_ and *c*_3_, respectively; DSB_1_ and DSB_2_ are rejoined with rates λ_1_ and λ_2_, respectively; DSB_3_ are unrejoinable.DSB_1_ and DSB_2_are “fixed” to become DSB_3_with rates ε_1_ and ε_2_, respectively. DSB_1_ interact with each other (by quadratic mis-rejoining) with rate η_1_, and DSB_2_ interact with each other with rate η_2_. DSB_1_ interact with DSB_2_ with rate η_1,2_. Details are described in the main text.

**Fig 2:**
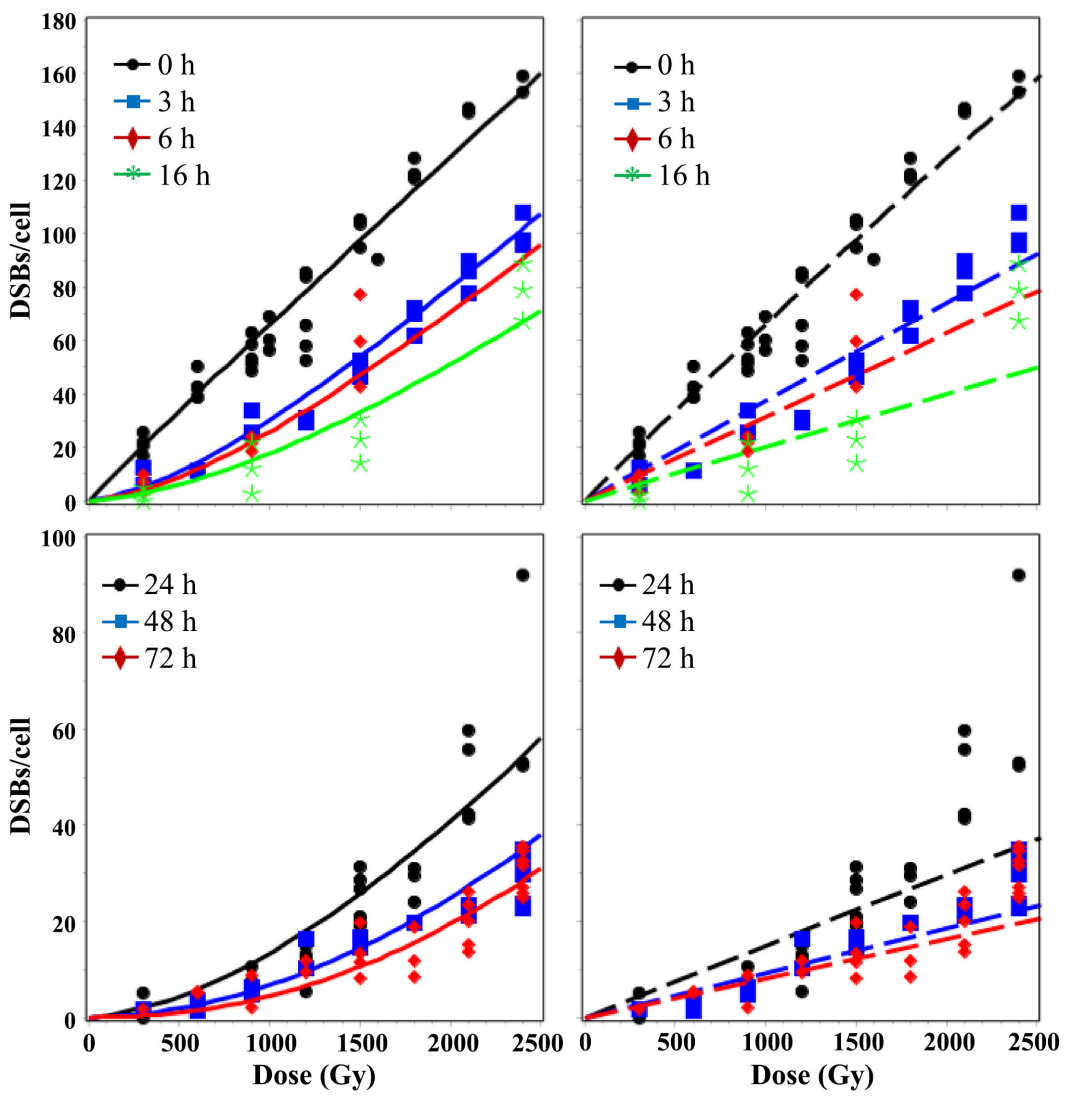
Dose dependence of best-fit model predictions (curves) for 30 MeV electron data (symbols). Solid lines = RD model, dashed lines = TLK model. The legend indicates times after HDR single-dose irradiation when DSBs were measured. In this and the following figures, the left-most panels compare both models, the middle panels show the RD model only, and the right-most panels show the TLK model only.

**Fig 3:**
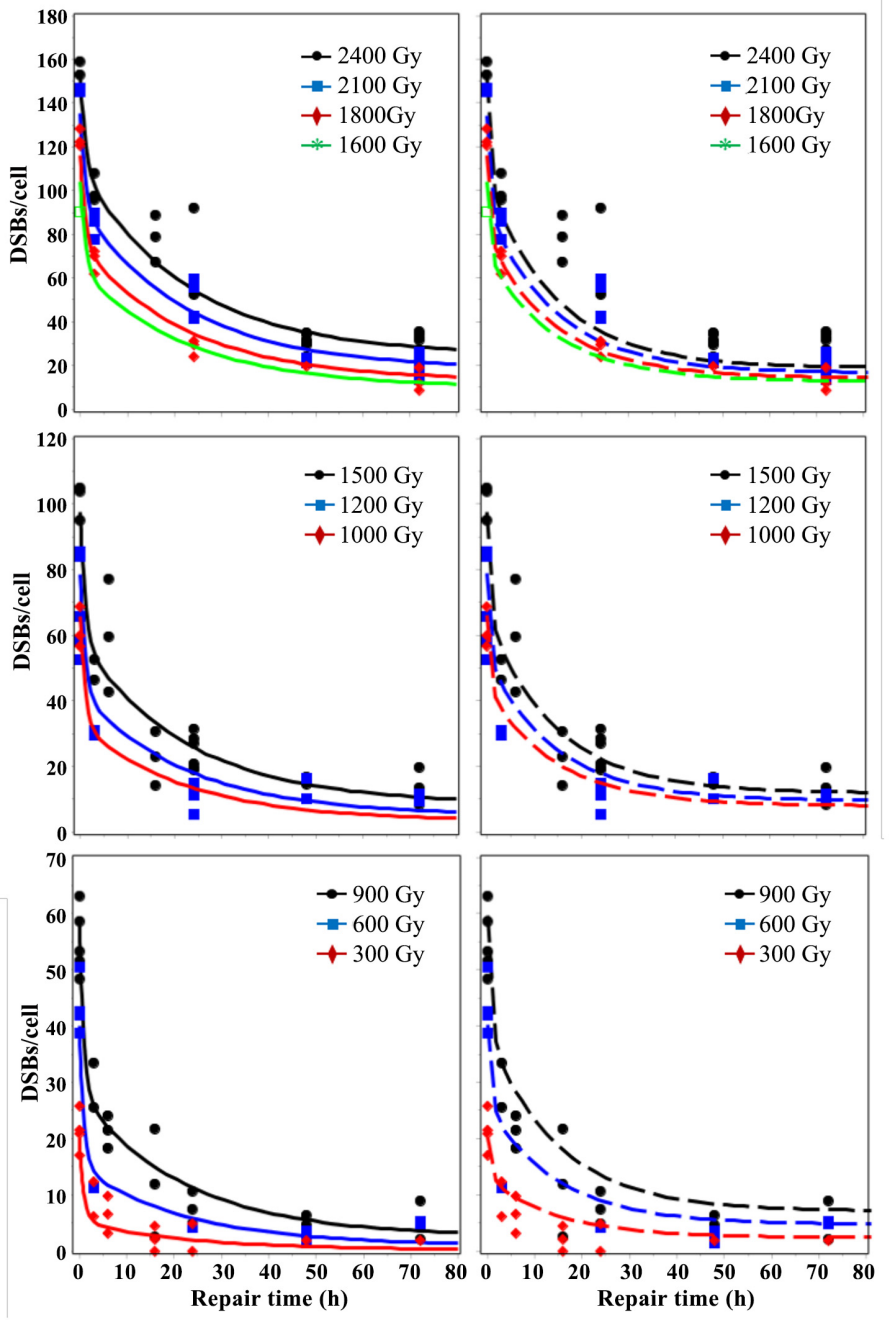
Time dependence of best-fit model predictions (curves) for 30 MeV electron data (symbols). Solid lines = RD model, dashed lines = TLK model. The legend indicates radiation doses (HDR, single-dose).

**Fig 4:**
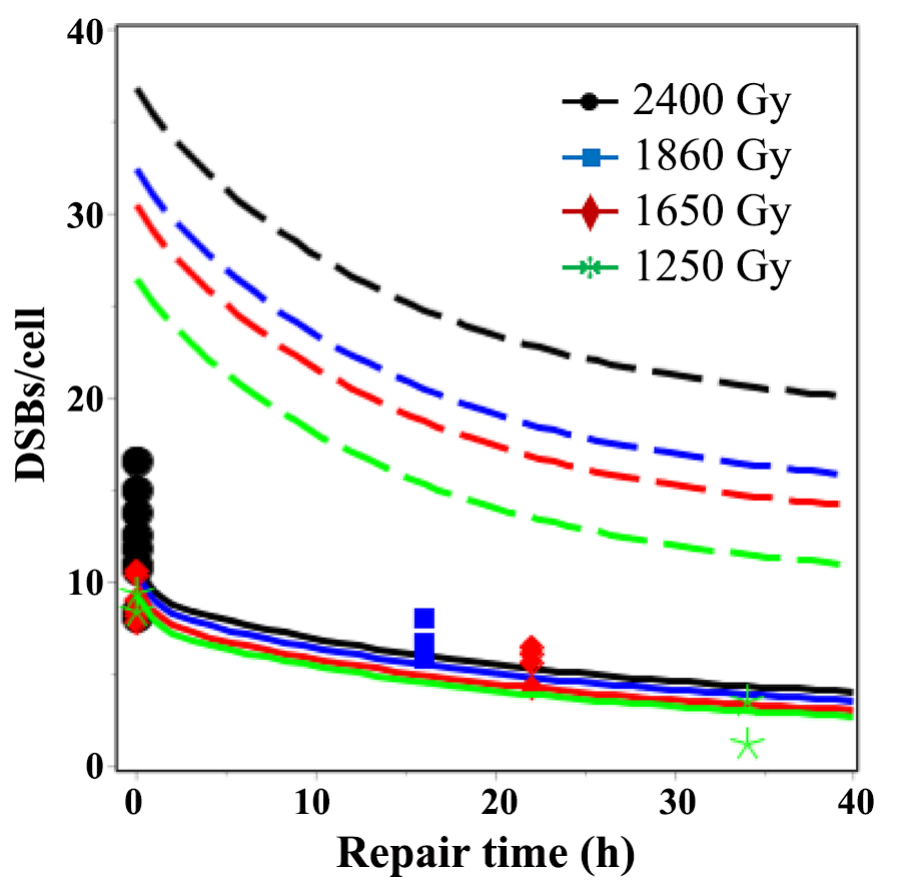
Time dependence of best-fit model predictions (curves) for LDR γ-ray data (symbols). Solid lines = RD model, dashed lines = TLK model. The legend indicates radiation doses.

**Fig 5:**
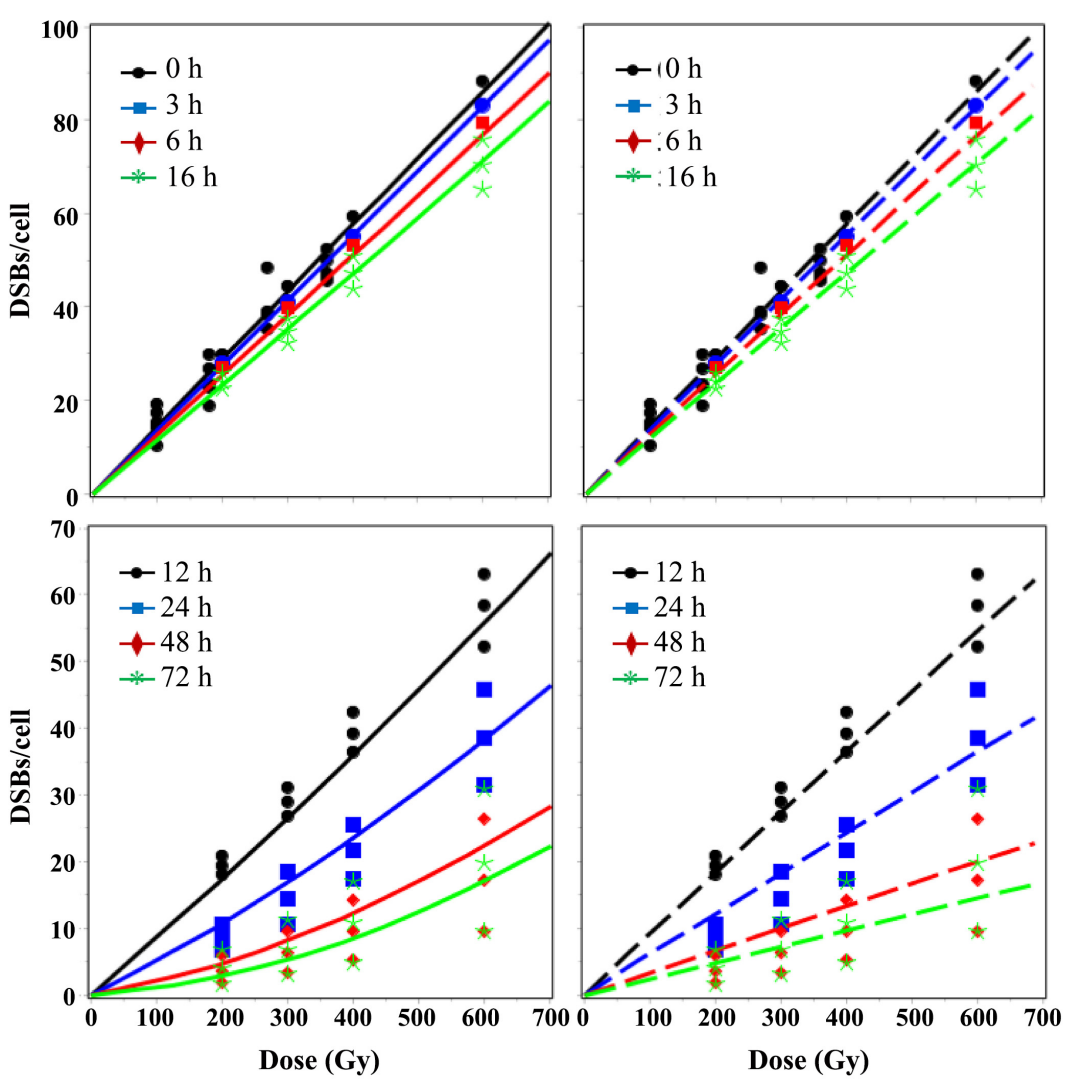
Dose dependence of best-fit model predictions (curves) for α-particle data (symbols). Solid lines = RD model, dashed lines = TLK model. The legend indicates times after HDR single-dose irradiation when DSBs were measured.

**Fig 6:**
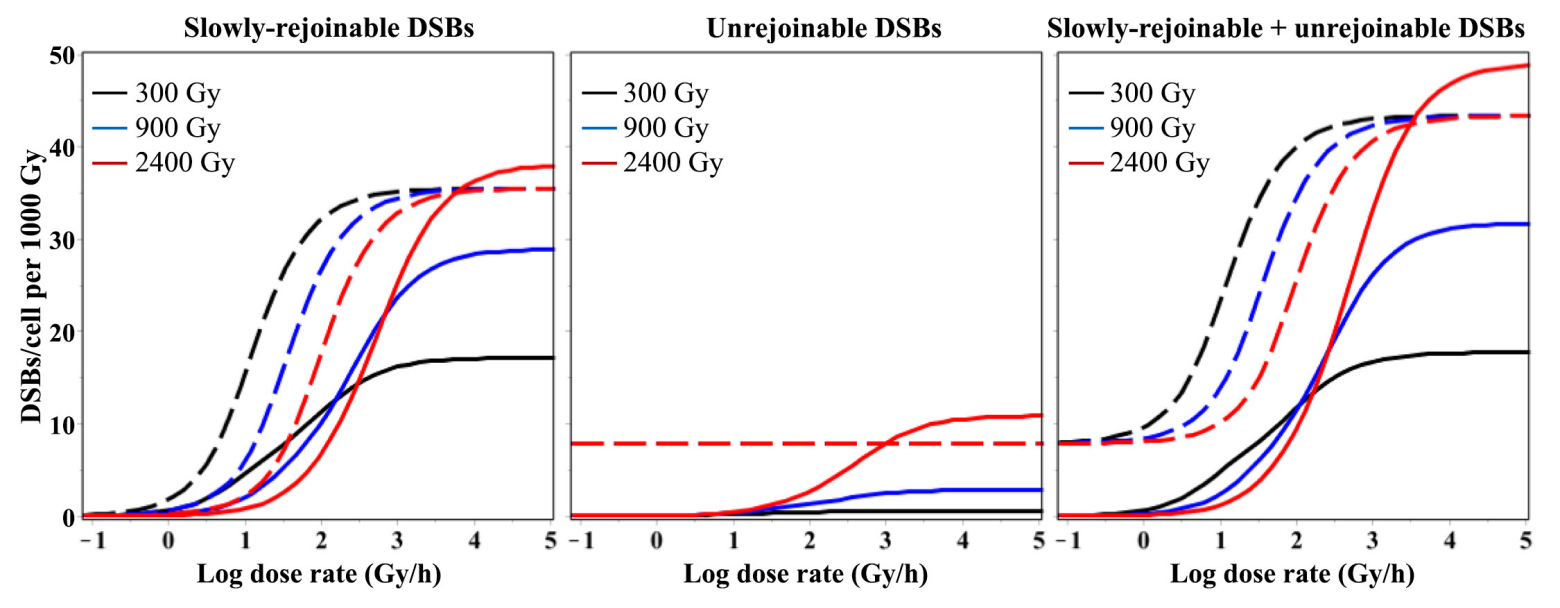
Dose rate dependence of best-fit model predictions for sparsely-ionizing radiation. Solid lines = RD model, dashed lines = TLK model. The legend indicates radiation doses.

This methodology was applied by Frankenberg-Schwager et al. to study DSB rejoining after sparsely-ionizing or densely-ionizing radiation under non-growth conditions, which allowed rejoining to occur, but prevented confounding of the results by cell proliferation. Here, we analyzed data sets for the following irradiation scenarios:

1. Sparsely-ionizing 30 MeV electrons, delivered at a high dose rate (7800 Gy/h), either as a single dose (300–2400 Gy) followed by rejoining time of 0–72 h, or as split doses (900–2400 Gy/dose) separated by a 16–48 h interval and followed by rejoining time of 0–24 h [38]. These data were taken from Figures 3–7 of reference [38]. The linear energy transfer (LET) was approximately 0.21 keV/μm.
2. Single doses (1250–2400 Gy) of sparsely-ionizing ^60^Co γ-rays delivered at a low dose rate (approximately 33 Gy/h) followed by rejoining time of 0–36 h [39]. These data were taken from Figures 1–2 of reference [39]. The LET was approximately 0.24 keV/μm.
3. Single doses (100–600 Gy) of densely-ionizing 3.5 MeV α-particles delivered at a high dose rate (1400 Gy/h) followed by rejoining time of 0–72 h [40, 41]. These data were taken from Figure 1 of reference [40] and Figure 2 of reference [41]. The LET was approximately 113 keV/μm.

For convenience, we classified the data as high dose rate (HDR, data sets 1 and 3) or low dose rate (LDR, data set 2) exposures. The data points, which were presented graphically in the cited publications by Frankenberg-Schwager et al. were digitized using GetData Graph Digitizer software (http://www.getdata-graph-digitizer.com/). In some cases (particularly for data set 3), not all experimentally measured data points were published: instead, only the mean and standard error were reported. In this situation we used a conservative assumption that the number of data points was three, and assigned values to these points so that the reported mean and standard error would be reproduced: one point was set equal to the mean, and the other two were symmetrically positioned around it at an appropriate distance to mimic the standard error. The total number of data points in the three data sets analyzed here was 278: 186 for sparsely-ionizing and 92 for densely-ionizing radiations. The values of these data points are provided in the S1 Appendix.

### Mathematical models

In the proposed RD model (schematically summarized in Fig 1), radiation (with dose rate R) produces three DSB classes (DSB_1_, DSB_2_, DSB_3_) with yields (per unit dose) of *k*_1_, *k*_2_ and *k*_3_, respectively. Quickly-rejoinable DSBs (DSB_1_) and slowly-rejoinable DSBs (DSB_2_) are rejoined with rates *v*_1_ and *v*_2_, respectively; DSB_3_ are unrejoinable. Radiation converts DSB_1_ to DSB_2_ and DSB_2_ to DSB_3_ with rates proportional to parameters *q*_1_ and *q*_2_, respectively. The model contains seven parameters: *k*_1_, *k*_2_, *k*_3_, *v*_1_, *v*_2_, *q*_1_ and *q*_2_. These assumptions are mathematically represented by the following system of differential equations:

$$\begin{array}{c} \frac{d{\text{DSB}}_{1}\left(t\right)}{dt}=k_{1}\times \text{R}-\left(v_{1}+q_{1}\times \text{R}\right)\times {\text{DSB}}_{1}\left(t\right);\\ \frac{d{\text{DSB}}_{2}\left(t\right)}{dt}=k_{2}\times \text{R}-\left(v_{2}+q_{2}\times \text{R}\right)\times {\text{DSB}}_{2}\left(t\right)+\;q_{1}\times \text{R}\times {\text{DSB}}_{1}\left(t\right);\\ \frac{d{\text{DSB}}_{3}\left(t\right)}{dt}=\;k_{3}\times \text{R}\;+\;q_{2}\times \text{R}\times {\text{DSB}}_{2}\left(t\right) \end{array}$$ (1)

These equations are analytically solvable, and the solutions are provided in the S2 Appendix for each DSB class at time T after a single radiation dose *D* is delivered with dose rate R. Linear quadratic (LQ) approximations to these solutions (which are computationally convenient and can be compared to analogous approximations of other kinetic models [46, 47]) are also included in the S2 Appendix.

In the TLK model [34] (schematically summarized in Fig 1), radiation also produces three DSB classes with yields (per unit dose) of *c*_1_, *c*_2_ and *c*_3_, respectively; DSB_1_ and DSB_2_ are rejoined with rates λ_1_ and λ_2_, respectively; DSB_3_ are unrejoinable. DSB_1_ and DSB_2_ are “fixed” to become DSB_3_ with rates ε_1_ and ε_2_, respectively. DSB_1_ interact with each other (by quadratic mis-rejoining) with rate η_1_, and DSB_2_ interact with each other with rate η_2_. DSB_1_ interact with DSB_2_ with rate η_1,2_. The model contains ten parameters: *c*_1_, *c*_2_, *c*_3_, λ_1_, λ_2_, ε_1_, ε_2_, η_1_, η_2_, and η_1,2_. These assumptions are represented by the following system of differential equations:

$$\begin{array}{c} \frac{d{\text{DSB}}_{1}\left(t\right)}{dt}=c_{1}\times \text{R}-\left(\varepsilon_{1}+\lambda_{1}\right)\times {\text{DSB}}_{1}\left(t\right)-\left(\eta_{1}\times {\text{DSB}}_{1}\left(t\right)+\eta_{1,2}\times {\text{DSB}}_{2}\left(t\right)\right)\times {\text{DSB}}_{1}\left(t\right);\\ \frac{d{\text{DSB}}_{2}\left(t\right)}{dt}=c_{2}\times \text{R}-\left(\varepsilon_{2}+\lambda_{2}\right)\times {\text{DSB}}_{2}\left(t\right)-\left(\eta_{2}\times {\text{DSB}}_{2}\left(t\right)+\eta_{1,2}\times {\text{DSB}}_{1}\left(t\right)\right)\times {\text{DSB}}_{2}\left(t\right);\\ \frac{d{\text{DSB}}_{3}\left(t\right)}{dt}=c_{3}\times \text{R}\;+\varepsilon_{1}\times {\text{DSB}}_{1}\left(t\right)+\;\varepsilon_{2}\times {\text{DSB}}_{2}\left(t\right) \end{array}$$ (2)

Here, we have included a term for direct yield of unrejoinable DSBs (parameter *c*_3_) for completeness, because although this term was absent from the original TLK model [34], it was introduced in subsequent similar formalisms [48]. There is no analytical solution to Eq 2.

### Model fitting procedure

The RD and TLK models were fitted to data by maximizing the log likelihood, using optimization routines in Maple 17® software. All sparsely-ionizing radiation data sets (HDR, single and split doses, and LDR, single doses) were fitted together to assess how well can each model describe all of these types of exposures using one set of parameters. Although two distinct types of sparsely-ionizing radiation (30 MeV electrons and ^60^Co γ-rays) were used to produce these data sets [38, 39], their biological effects were likely to be similar. Densely-ionizing radiation data (HDR, single doses only) [40, 41] were fitted separately. All parameters were restricted to ≥ 0 to maintain mechanistic plausibility.

We assumed a Gaussian error distribution with constant variance for all data points. This assumption was reasonable because the errors introduced during measurement of the data (DSB/cell yields) were not estimated explicitly [38] and were likely to be the same for all data points. The log likelihood function (LL_M,i_) under assumption of constant variance, is described by the following equation:

$$LL_{M,i}=-\frac{1}{2}N_{\left(i\right)}\times \left(ln\left[\sum_{j}\left({\left[P_{M,i,\left(j\right)}-O_{i,\left(j\right)}\right]}^{2}/N_{\left(i\right)}\right)\right]+\;\text{ln}\left(2\pi \right)\;+1\right)$$ (3)

Here, the index M represents either the RD or the TLK model, *i* represents the data set (either sparsely-ionizing or densely-ionizing radiation), N_(i)_ is the total number of data points for the *i*-th data set, P_M,i,(j)_ are predictions of the M-th model at the *j*-th data point, and O_i,(j)_ are observed data values. The constant term ln(2π)+1 was included for completeness, but had no effect on the comparison of model performances and on parameter estimation.

Analytic solutions for the RD model were used to calculate P_M,i,(j)_, and the likelihood function (Eq 3) was optimized by a sequential quadratic programming (SQP) algorithm [49] implemented in Maple 17® software. The probability of finding the global maximum (rather than local maxima) was enhanced by using 100 random initial conditions for the model parameters. For the TLK model, only numerical solutions could be obtained. Consequently, a customized optimization procedure (described in the S2 Appendix), also with 100 random initial conditions, written in Maple 17® was used for fitting the TLK model to data.

Absolute goodness of fit (GOF) for the analyzed models under assumption of constant variance was assessed by exploratory data fitting using three methods: (1) visual inspection of model fits and the data; (2) calculation of the coefficient of determination, R^2^; (3) linear regression of model predictions P_M,i,(j)_ vs. data points O_i,(j)_. Model fits were assumed to have no gross systematic deviations from the data when the 95% confidence intervals (CIs) for the regression of model predictions vs. data included 0 for the intercept and 1 for the slope.

Additional exploratory calculations showed that an alternative assumption of error magnitudes proportional to O_i,(j)_ values, which resulted in replacement in Eq 3 of the term [P_M,i,(j)_−O_i,(j)_]^2^ by the term [P_M,i,(j)_−O_i,(j)_]^2^/ O_i,(j)_^2^, reduced GOF for both the RD and TLK models because the data points at low doses, which had small values of O_i,(j)_, affected the fit more strongly than other points.

### Estimation of model parameter uncertainties

Uncertainties (95% CIs) for best-fit model parameter values were estimated by profile likelihood [50] as follows: 10,000 Monte-Carlo-generated parameter values in the vicinity of the best-fit values were used to estimate the critical contour of the log likelihood function, which is based on the asymptotic X^2^ behavior of the log likelihood distribution.

### Information theoretic model selection

Ranking of models by relative support from the data, taking into account sample size and number of parameters, can be based on the Akaike information criterion with sample size correction (AICc). AICc has gained popularity for this purpose in various fields [51, 52]. For comparing non-linear models, AICc is preferable to methods that rely on reduced X^2^ or R^2^ [53–56]. One of the most useful features of AICc is that it allows the evidence for structurally distinct models to be compared, without the need for models to be “nested” or to belong to the same class. The model that loses the least amount of Kullback–Leibler information relative to other compared models has the lowest AICc value and is considered as best-supported by the data at hand. The equation for AICc for the M-th model on the *i*-th data set (AICc_M,i_) is given below, where *K*_M_ is the number of adjustable parameters and LL_M,i_ is the maximized log-likelihood value (calculated using Eq 3):

$$AICc_{M,i}=-2\;LL_{M,i}\;+\;2\;K_{M}\;+\;2\;K_{M}\;(K_{M}\;+\;1)/(N_{\left(i\right)}-K_{M}-1)$$ (4)

The likelihood of the M-th model relative to other tested model(s), called the evidence ratio (*ER*_M,i_), can be expressed as:

$$ER_{M,i}=\text{exp}\left[-\frac{1}{2}\Delta AICc_{M,i}\right]\;,\;\text{where}\;\Delta AICc_{M,i}=\;AICc_{M,i}-AICc_{min,i}$$ (5)

Here, AICc_min,i_ is the lowest AICc value generated by the set of models being compared. If ΔAICc_M,i_ > 6, then the evidence ratio *ER*_M,i_ becomes < 0.05, suggesting that the M-th model has much poorer support from the data than the best-supported model.

In contrast to the AICc, the Bayesian information criterion BIC [57] does not adjust for sample size effects [52]. The F-test requires arbitrary assumptions about test type (forward, backward or stepwise) and about the test significance threshold (α-value) [52], and provides no readily-interpretable information on relative support for each model from the data. Consequently, we believe that AICc is the most useful and convenient tool for selecting among plausible mechanistic models using DSB rejoining data.

## Results

### Comparison of model performance

Best-fit predictions of the RD and TLK models after single-dose exposures are compared with the data in Figs 2–5. Visual inspection clearly shows that at long times (≥ 24 h) after sparsely-ionizing or densely-ionizing radiation only the RD model reproduced the upwardly-curving dose responses for remaining unrejoined DSBs, whereas the TLK model predicted linear dose responses and underestimated the data. For example, the mean measured number of DSBs/cell 72 hours after 2400 Gy of HDR 30 MeV electrons was 30.2 (range: 24.7–35.6). The corresponding best-fit prediction from the RD model was 28.4, whereas the TLK model predicted 19.5 (Fig 2). The mean measured number of DSBs/cell 72 hours after 600 Gy of α-particles was 20.1 (range: 9.6–30.9). The corresponding best-fit prediction from the RD model was 17.0, whereas the TLK model predicted 14.5 (Fig 5). Therefore, the tendency of the TLK model to underestimate the data at long times and high doses was evident for both sparsely- and densely-ionizing radiations, although it was more clear with the former. The number of split-dose data points (10) was too small for robust conclusions (i.e. both models visually approximated these data reasonably), and consequently we did not show the fits to these data graphically.

Although the number of HDR data points (157) for sparsely-ionizing radiation was much larger than the number of LDR points (29), the RD model reasonably described DSB yields at both high and low dose rates using one set of parameters (Fig 4). In contrast, the TLK model was unable to do so: its fit was dominated by HDR data and LDR data were strongly overestimated (Fig 4). For example, the mean measured number of DSBs/cell 22 hours after 1650 Gy of LDR γ-rays was 5.6 (range: 4.2–6.4). The corresponding best-fit prediction from the RD model was 4.0, whereas the TLK model predicted 16.8 (Fig 4).

These visually-apparent differences in model performance were quantified by relative and absolute GOF assessments (Tables 1 and 2). The RD model described the sparsely-ionizing radiation data dramatically better than the TLK model: the sum of squared deviations from the data was 3.1–fold lower for the RD model, and this translated into a massive difference in support of 217 AICc units (Table 1).

**Table 1:** Information theoretic performance assessment (by ΔAICc) and best-fit parameter values for the RD and TLK models. ΔAICc = 0 suggests the strongest support from the data for the given model among all tested models, whereas ΔAICc > 6 suggests poor support. Parameter meanings are provided in the caption to Fig 1 and in the main text.

Radiation	Model	ΔAICc	Best-fit parameter values (95% CIs)
Sparsely-ionizing	RD	0.0	*k*_1_ = 6.08 (5.83,6.23)×10^−2^ Gy^-1^ *k*_2_ = 7.83 (3.52,10.5)×10^−3^ Gy^-1^ *k*_3_ = 0 Gy^-1^*v*_1_ = 1.07 (0.73,2.12) h^-1^ *v*_2_ = 4.44 (3.65,5.28)×10^−2^ h^-1^ *q*_1_ = 1.22 (1.03,1.41)×10^−3^ Gy^-1^*q*_2_ = 2.67 (2.02,3.01)×10^−4^ Gy^-1^
	TLK	216.9	*c*_1_ = 2.49 (1.75, 3.52)×10^−2^ Gy^-1^ *c*_2_ = 35.5 (18.6, 41.1)×10^−3^ Gy^-1^*c*_3_ = 7.86 (4.68, 9.98)×10^−3^ Gy^-1^ λ_1_ = 1.32 (0.32, 5.12) h^-1^ λ_2_ = 6.72 (3.18, 9.32)×10^−2^ h^-1^ε_1_ = 0 h^-1^ ε_2_ = 0 h^-1^ η_1_ = 0 h^-1^ η_2_ = 0 h^-1^ η_1,2_ = 0 h^-1^
Densely-ionizing	RD	0.0	*k*_1_ = 0 Gy^-1^*k*_2_ = 145 (143, 147)×10^−3^ Gy^-1^ *k*_3_ = 0 Gy^-1^*v*_1_ = 0 h^-1^ *v*_2_ = 4.61 (3.97, 5.22)×10^−2^ h^-1^ *q*_1_ = 0 Gy^-1^*q*_2_ = 6.29 (5.04, 8.11)×10^−4^ Gy^-1^
	TLK	14.4	*c*_1_ = 0 Gy^-1^ *c*_2_ = 125 (117, 133)×10^−3^ Gy^-1^ *c*_3_ = 19.7 (12.0, 26.3)×10^−3^ Gy^-1^ λ_1_ = 0 h^-1^ λ_2_ = 4.60 (3.88, 5.26)×10^−2^ h^-1^ε_1_ = 0 h^-1^ ε_2_ = 0 h^-1^ η_1_ = 0 h^-1^ η_2_ = 0 h^-1^ η_1,2_ = 0 h^-1^

**Table 2:** GOF assessment for the RD and TLK models. R^2^ = coefficient of determination. The intercept (*i*) and slope (*s*) of linear regression of model predictions vs. observed data point values were used to assess GOF. Systematic deviations of predictions from the data (marked in bold font) are suggested if the 95% CIs (shown in parentheses) of the intercept do not include zero and/or if the 95% CIs of the slope do not include unity.

Radiation	Model	GOF assessment:		
		HDR, one dose	HDR, split doses	LDR, one dose
Sparsely-ionizing	RD	R^2^ = 0.951; i = -1.02 (-2.98, 0.93); s = 1.02 (0.98, 1.06)	R^2^ = 0.961; i = 0.84 (-9.70, 11.38); s = 0.96 (0.80, 1.12)	R^2^ = 0.660; i = 1.52 (-0.62, 3.66); s = 0.88 (0.63, 1.13)
	TLK	R^2^ = 0.910; i = 0.54 (-2.06, 3.15); s = 1.04 (0.98, 1.09)	R^2^ = 0.903;i = 3.47 (-12.84, 19.77); s = 0.99 (0.72, 1.25)	R^2^ = 0.681; i = -0.23 (-2.72, 2.26); s = **0.32 (0.23, 0.40)**
Densely-ionizing	RD	R^2^ = 0.958; i = -0.06 (-1.63, 1.51); s = 1.00 (0.96, 1.05)		
	TLK	R^2^ = 0.955; i = -1.11 (-2.77, 0.54); s = 1.03 (0.98, 1.07)		

On the smaller α-particle data set, which included only HDR exposures, the difference in model performances was reduced, but the RD model was still strongly favored: by 14 AICc units (Table 1). R^2^ was higher and 95% CIs for the intercept and slope of linear regression of model predictions vs. observed data point values were often narrower for the RD model vs. the TLK model for HDR data (Table 2). Systematic deviations of predictions from LDR data were suggested for the TLK model, for which the regression slope CIs did not include unity, but no such deviations were detected for the RD model (Table 2).

The AICc scale being logarithmic, the relative support from the sparsely-ionizing data for the TLK model vs. the RD model (i.e. the evidence ratio, Eq 5) is exp[–217/2], which is zero for all practical purposes. For densely-ionizing radiation data, the TLK model evidence ratio is exp[–14/2], or approximately 1/1400. These marked differences in support are dominated by the differences in the log likelihoods (Eq 3) of the RD and TLK models (105 units for sparsely-ionizing and 3.5 units for densely-ionizing radiation data), rather than by a mere penalization for extra parameters. In fact, even if the TLK model were artificially favored, for example, by fixing three of its parameters (η_1_, η_2_, and η_1,2_) at best-fit values, thereby reducing the number of adjustable parameters to the same number as in the RD model (7), the RD model would remain the best-ranked model by a still very comfortable margin of 210 AICc units for sparsely-ionizing and 7.1 units for densely-ionizing radiation data.

However, it is important to note that the TLK model performed much better than its predecessor–the LPL formalism [37]. When applied to DSB rejoining data, the LPL model contains two classes of DSBs (rejoinable and unrejoinable) and 4 adjustable parameters: the yields of each DSB class per unit dose, and coefficients for linear and quadratic DSB rejoining processes. On sparsely-ionizing radiation data, this simpler model had dramatically less support than the TLK and RD models (by 314 and 531 AICc units, respectively). R^2^ for the LPL model on HDR single-dose data was only 0.25, whereas it was 0.91 and 0.95 for the TLK and RD models, respectively (Table 2). On densely-ionizing data, the LPL model was worse than the TLK and RD models by 204 and 218 AICc units, respectively, and its R^2^ was 0.52 (vs. 0.96 for the TLK and RD models).

These results suggest that the TLK formalism, which contains three DSB classes, represents an important improvement over the LPL model with two classes. However, the RD model, which contains radiation-induced conversion of DSB classes, yields a further major improvement for the yeast data at hand. The improved performance of the RD model relative to the TLK model is caused specifically by the dose/dose rate dependence of conversion of DSB classes: if the conversion is allowed to occur without influence by radiation (i.e. the terms *q*_1_×R and *q*_2_×R in Eq 1 are replaced with *q*_1_ and *q*_2_, respectively), model performance plummets by 210 and 7 AICc units for sparsely-ionizing and densely-ionizing radiation, respectively. In other words, without radiation-induced conversion of DSB classes, the RD model performs equivalently to the TLK model.

### RD model parameter values

The best-fit RD model parameter values (Table 1) suggested that sparsely-ionizing radiation directly induced approximately 8 times more quickly-rejoinable than slowly-rejoinable DSBs (based on the parameter ratio *k*_1_/*k*_2_). The direct yield of unrejoinable DSBs was not detectable: parameter *k*_3_ had a best-fit value of zero. Consequently, the suggested interpretation is that unrejoinable DSBs were produced only indirectly by conversion of slowly-rejoinable DSBs with a rate proportional to the radiation dose rate and to parameter *q*_2_. Conversion of quickly-rejoinable DSBs (with a rate proportional to the radiation dose rate and to parameter *q*_1_) was an important indirect source of slowly-rejoinable DSBs. The rejoining rates for these two DSB classes differed by a factor of 24 (based on the parameter ratio *v*_1_/*v*_2_).

In contrast, the RD model suggested that densely-ionizing α-particles directly produced only slowly-rejoinable DSBs; whereas the direct yields of quickly-rejoinable and unrejoinable DSBs were not detectable: parameters *k*_1_ and *k*_3_ had best-fit values of zero (Table 2). The yield of slowly-rejoinable DSBs per unit dose was approximately 18.5–fold higher than for sparsely-ionizing radiation (based on the ratio of parameter *k*_2_ values). However, their rejoining rate (parameter *v*_2_) was the same for both sparsely-ionizing and densely-ionizing radiation (Table 1). The rate (per unit dose) of conversion of slowly-rejoinable DSBs to unrejoinable ones was approximately 2–fold higher for α-particles vs. sparsely-ionizing radiation (Table 1).

## Discussion

We analyzed large published data sets on DSB rejoining in yeast (*Saccharomyces cerevisiae*) exposed to sparsely-ionizing and densely-ionizing radiation [38–41] to enhance the understanding of dose/dose rate dependences of radiation-induced DSB rejoining kinetics. Specifically, we tested the hypothesis that the fraction of slowly-rejoinable/unrejoinable DSBs increases with increasing dose/dose rate. This hypothesis was implemented in a new RD model, whose performance was compared to that of the established TLK model [34].

The strengths of the current study include rigorous information theoretic comparison of the performances of two models with different assumptions about the mechanisms of DSB rejoining, using extensive data sets which include different radiation types, doses, dose rates, and rejoining times. The weaknesses include the use of *S*. *cerevisiae* (rather than mammalian cell) data. Yeast data are useful because DSB rejoining was measured in the dose range relevant for clonogenic cell survival [45], but some aspects of DSB rejoining in yeast and mammalian cells are different, e.g. because *S*. *cerevisiae* relies on homologous recombination to a greater extent than many other organisms [58, 59], and chromatin structure is also different in yeast and mammals [60]. The numbers of radiation-induced DSBs per cell at the mean lethal dose for yeast and mammalian cells are comparable [60, 61]. However, because the yeast genome is much smaller than mammalian genomes [62], radiation doses used for yeast studies (≥ 300 Gy of sparsely-ionizing radiation) are much higher than those relevant for clonogenic survival of mammalian cells (< 20 Gy). This difference in absolute dose magnitudes may have an effect on DSB rejoining kinetics which is independent of the number of DSBs per cell, but dependent on other factors such as the yields of reactive oxygen and nitrogen species (ROS and RNS). For example, radiation-induced oxidants reduce chromosomal movements by affecting the meiotic actin cytoskeleton, thereby potentially preventing unfavorable chromosome interactions [63]. In addition, at radiation doses relevant for yeast survival, cellular enzyme inactivation and changes in cell membrane integrity and permeability may play more important roles in cell death [64], than at the much lower doses relevant for mammalian cell survival.

Despite these potential drawbacks, we believe that the current study provides potentially clinically-relevant insight into mechanistic modeling of DSB rejoining. In particular, because the TLK and RD models are based on different mechanistic assumptions, comparison of their performances on the same data enabled us to make the following observations about which putative mechanisms are most (or least) useful for describing DSB rejoining kinetics.

The TLK model contains 3 parameters (η_1_, η_2_, and η_1,2_) for quadratic interactions between different DSBs within/between DSB classes (Fig 1). If some/all of these parameters have positive values, the model structure (Eq 2) implies that, as the dose of HDR radiation (and hence the yield of DSBs just after irradiation) increase, the rate of DSB removal increases because quadratic interactions between DSBs become more frequent. Consequently, the quadratic interactions mechanism predicts that the fraction of DSBs which remain unrejoined at a given time after irradiation should decrease with increasing radiation dose. The dose response for unrejoined DSBs at a given time after irradiation is, therefore, predicted to be downwardly-curving (i.e. to have a negative second derivative).

In contrast, the data analyzed here suggest the opposite pattern: at higher doses, the fraction of DSBs which remain unrejoined at each measured time after irradiation increases, rather than decreases (Figs 2, 3 and 5) [61]. Consequently, the observed dose response for unrejoined DSBs at a given time after irradiation is upwardly-curving (i.e. has a positive second derivative). This disparity between predictions and observations explains why the best-fit values of parameters η_1_, η_2_, and η_1,2_ in the TLK model became zero (Table 1): when quadratic interactions between DSBs do not occur, the TLK model predicts a linear dose response for unrejoined DSBs (Figs 2 and 5), which is closer to the data than a downwardly-curving one.

The mechanism of DSB fixation, which is also present in the TLK model, could not be detected in the data analyzed here: the best-fit values of parameters ε_1_ and ε_2_ were zero (Table 1). If either/both of these parameters were restricted to positive values, the fit quality did not change substantively, and the main outcome was reduction of the best-fit value of direct induction of unrejoinable DSBs (parameter *c*_3_). In other words, the TLK model relied on unrejoinable DSBs (produced directly by radiation and/or by fixation of rejoinable DSBs) to describe the HDR data for both sparsely-ionizing and densely-ionizing radiation (Table 1). If this mechanism was turned off by setting parameter *c*_3_ to zero, TLK model performance strongly worsened: by 7.6 AICc units for sparsely-ionizing and by 17.7 units for densely-ionizing radiation.

An important drawback of the TLK model’s reliance on unrejoinable DSBs to approximate HDR data was that the best-fit yield of such DSBs (parameter *c*_3_) was too high to describe LDR data, leading to overestimation of these data (Fig 4). In other words, in the context of the TLK model, the observed DSB data at long times after HDR sparsely-ionizing radiation exposure appeared to be inconsistent with the data after LDR exposure.

These results are in agreement with previous analyses which suggested that the RMR model does not describe yeast DSB rejoining data well [65]. The current study shows that the TLK model, which is more flexible than the RMR model because it allows for different DSB classes, achieved decent absolute GOF (Table 2). However, the TLK model performed very poorly relative to the RD model on the data at hand because it lacked the proposed mechanism of radiation-induced conversion of DSB classes.

In contrast, the proposed mechanism of radiation-dependent conversion of DSB classes present in the RD model (Fig 1) allowed the upwardly-curving dose response shape for DSBs left unrejoined after HDR radiation to be reproduced (Figs 2 and 5). There was also no inconsistency between HDR and LDR data in the context of the RD model (Figs 2–4): both data sets were described with adequate GOF (Table 2) using one set of best-fit parameters. These results were obtained thanks to the fact that the RD model predicts that, at increasing doses/dose rates, DSB rejoining is increasingly impeded because more and more quickly-rejoinable DSBs are converted to slowly-rejoinable DSBs, some of which are in turn converted to unrejoinable ones.

The qualitative differences in dose and dose rate dependences of DSB rejoining predicted by the RD and TLK models (using best-fit parameter values) can be visualized in Fig 6. The TLK model predicts that the yield of unrejoinable DSBs per unit dose is independent of both dose and dose rate, whereas the yield of slowly-rejoinable DSBs depends on dose rate but not on dose. The RD model, however, predicts that the yields of both slowly-rejoinable and unrejoinable DSBs depend on both dose and dose rate (Fig 6).

Our analysis suggests that the proposed mechanism of radiation-dependent conversion of DSB classes strongly enhanced the ability of kinetic models to describe DSB rejoining in yeast. In contrast, the analyzed data did not support the notion (represented in the TLK model) that interactions between different DSBs constitute an important route of DSB removal. However, interactions between DSBs which lead to transition into a different class of DSB would still be compatible with the data. Our analysis also suggests that the time needed to rejoin a particular DSB depends mainly on the properties of this DSB and/or on radiation damage to nearby chromatin. These factors can be represented by the generic term “DSB complexity”, which depends not only on radiation type, but also on dose and dose rate.

The previously mentioned GLOBLE model [17, 28] provides a conceptually similar interpretation and also predicts that the fraction of slowly-rejoinable/unrejoinable DSBs should increase with radiation dose. Specifically, the GLOBLE model assumes that the genome is organized into chromatin loops, and that the presence of one or multiple DSBs inside a given loop is associated with different damage severity and rejoining rates. The GLOBLE approach has been applied to mammalian cell data, but it is not clear whether its assumptions are applicable to yeast because chromatin organization in yeast differs from that in mammals [66, 67].

In contrast, the RD model can be viewed as a more generalized approach where the nature of DSB classes and the mechanisms of conversion between them are not specifically tied to chromatin structure. For example, when repair enzymes encounter a complex DSB, and/or are oxidatively damaged during the repair process (which is more likely to occur at higher radiation doses/dose rates), rejoining of the DSB may become stalled. Conceptually similar explanations were considered by the authors of the data sets analyzed here, but were never implemented as a mathematical model [68]. Such explanations, as well as those based on chromatin structure, are consistent with the RD formalism.

The LQ approximation to RD model solutions for the number of DSBs which remain unrejoined at long (effectively infinite) rejoining times (Eqs. A3-4 of the S2 Appendix) provides additional insight into dose rate effects within the context of the RD model. At all dose rates, the linear DSB dose response component is composed of the yield of unrejoinable DSBs (parameter *k*_3_). The quadratic component is composed of the product of the yield of slowly-rejoinable DSBs (parameter *k*_2_) and the conversion coefficient for slowly-rejoinable to unrejoinable DSBs (parameter *q*_2_). This quadratic component is absent at low dose rates. Thus, lowering the dose rate reduces the quadratic dose response term, but the mechanistic interpretation of this phenomenon is not the same as in other kinetic models: in other models the quadratic term is reduced because, at low dose rates, most DSBs are rejoined before they can interact with each other; whereas in the RD model the quadratic term is reduced because, at low dose rates, most quickly-rejoinable DSBs are rejoined before radiation can convert them to slowly-rejoinable ones.

These conclusions are important for mechanistic understanding and quantitative modeling of DSB rejoining kinetics and have potential clinical relevance, e.g. for optimizing cancer radiotherapy. However, because we cannot exclude the possibility that the results of this study are specific to *S*. *cerevisiae*, additional testing of the RD model needs to be performed on mammalian cell data. Specifically, it remains to be determined what molecular mechanisms define the differences between DSB classes and explain radiation-induced conversion between them.

## Supporting Information

S1 Appendix (DOCX)

S2 Appendix (DOCX)
