# Supplementary material for: Mechanistic Modeling of Dose and Dose Rate Dependences of Radiation-Induced DNA Double Strand Break Rejoining Kinetics in Saccharomyces cerevisiae
Source: PLoS One. 2016 Jan 7;11(1):e0146407. doi: 10.1371/journal.pone.0146407 (PMC4711806; doi:10.1371/journal.pone.0146407)
Supplement: S1 Appendix — (DOCX) [file pone.0146407.s001.docx]

**ANALYZED Data sets**

Here, we analyzed data sets for the following irradiation scenarios:

(1) Sparsely-ionizing 30 MeV electrons, delivered at a high dose rate (7800 Gy/h), either as a single dose (300–2400 Gy) followed by rejoining time of 0–72 h, or as split doses (900–2400 Gy/dose) separated by a 16–48 h interval and followed by rejoining time of 0–24 h [[38](#_ENREF_38)]. These data were taken from Figures 3-7 of reference [[38](#_ENREF_38)]. The linear energy transfer (LET) was approximately 0.21 keV/μm. The data points (estimated DSB/cell) in this and the following data sets were digitized using GetData Graph Digitizer software (<http://www.getdata-graph-digitizer.com/>) as described in the main text.

Single doses:

| Rejoining  time (h) | Dose (Gy) | Estimated  DSB/cell |
| --- | --- | --- |
| 0 | 300 | 16.9 |
| 0 | 300 | 20.8 |
| 0 | 300 | 21.5 |
| 0 | 300 | 25.7 |
| 0 | 600 | 38.8 |
| 0 | 600 | 38.8 |
| 0 | 600 | 41.9 |
| 0 | 600 | 42.6 |
| 0 | 600 | 50.4 |
| 0 | 900 | 48.3 |
| 0 | 900 | 51.5 |
| 0 | 900 | 53.2 |
| 0 | 900 | 58.5 |
| 0 | 900 | 63.1 |
| 0 | 1000 | 56.4 |
| 0 | 1000 | 59.9 |
| 0 | 1000 | 68.7 |
| 0 | 1200 | 52.5 |
| 0 | 1200 | 58.1 |
| 0 | 1200 | 65.6 |
| 0 | 1200 | 83.9 |
| 0 | 1200 | 85.6 |
| 0 | 1500 | 94.8 |
| 0 | 1500 | 103.6 |
| 0 | 1500 | 105.0 |
| 0 | 1600 | 90.2 |
| 0 | 1800 | 120.5 |
| 0 | 1800 | 121.9 |
| 0 | 1800 | 128.3 |
| 0 | 2100 | 145.2 |
| 0 | 2100 | 147.0 |
| 0 | 2400 | 153.0 |
| 0 | 2400 | 158.9 |
| 3 | 300 | 6.0 |
| 3 | 300 | 12.3 |
| 3 | 600 | 11.3 |
| 3 | 900 | 25.4 |
| 3 | 900 | 33.5 |
| 3 | 1200 | 29.6 |
| 3 | 1200 | 31.0 |
| 3 | 1500 | 46.2 |
| 3 | 1500 | 52.5 |
| 3 | 1800 | 61.7 |
| 3 | 1800 | 69.8 |
| 3 | 1800 | 71.9 |
| 3 | 2100 | 77.9 |
| 3 | 2100 | 86.0 |
| 3 | 2100 | 89.9 |
| 3 | 2400 | 95.9 |
| 3 | 2400 | 97.6 |
| 3 | 2400 | 107.8 |
| 6 | 300 | 3.0 |
| 6 | 300 | 6.6 |
| 6 | 300 | 9.8 |
| 6 | 900 | 18.3 |
| 6 | 900 | 21.5 |
| 6 | 900 | 24.1 |
| 6 | 1500 | 42.6 |
| 6 | 1500 | 59.5 |
| 6 | 1500 | 76.9 |
| 16 | 300 | 0.0 |
| 16 | 300 | 2.0 |
| 16 | 300 | 4.4 |
| 16 | 900 | 2.5 |
| 16 | 900 | 11.8 |
| 16 | 900 | 21.7 |
| 16 | 1500 | 14.2 |
| 16 | 1500 | 22.7 |
| 16 | 1500 | 30.7 |
| 16 | 2400 | 67.2 |
| 16 | 2400 | 78.7 |
| 16 | 2400 | 88.5 |
| 24 | 300 | 4.9 |
| 24 | 300 | 0.0 |
| 24 | 600 | 4.2 |
| 24 | 900 | 4.9 |
| 24 | 900 | 7.4 |
| 24 | 900 | 10.6 |
| 24 | 1200 | 5.3 |
| 24 | 1200 | 11.3 |
| 24 | 1200 | 11.6 |
| 24 | 1200 | 12.3 |
| 24 | 1200 | 13.0 |
| 24 | 1200 | 14.8 |
| 24 | 1500 | 19.0 |
| 24 | 1500 | 19.7 |
| 24 | 1500 | 20.8 |
| 24 | 1500 | 26.8 |
| 24 | 1500 | 28.5 |
| 24 | 1500 | 31.4 |
| 24 | 1800 | 24.0 |
| 24 | 1800 | 29.3 |
| 24 | 1800 | 31.0 |
| 24 | 2100 | 41.2 |
| 24 | 2100 | 42.3 |
| 24 | 2100 | 55.7 |
| 24 | 2100 | 59.6 |
| 24 | 2400 | 52.2 |
| 24 | 2400 | 52.9 |
| 24 | 2400 | 92.0 |
| 48 | 300 | 1.8 |
| 48 | 600 | 1.4 |
| 48 | 600 | 1.8 |
| 48 | 600 | 3.5 |
| 48 | 900 | 4.6 |
| 48 | 900 | 6.3 |
| 48 | 1200 | 10.2 |
| 48 | 1200 | 16.2 |
| 48 | 1500 | 14.4 |
| 48 | 1500 | 15.5 |
| 48 | 1500 | 16.6 |
| 48 | 1800 | 19.7 |
| 48 | 2100 | 21.1 |
| 48 | 2100 | 23.3 |
| 48 | 2400 | 22.6 |
| 48 | 2400 | 23.6 |
| 48 | 2400 | 29.6 |
| 48 | 2400 | 30.7 |
| 48 | 2400 | 31.7 |
| 48 | 2400 | 34.2 |
| 48 | 2400 | 34.9 |
| 72 | 300 | 1.8 |
| 72 | 600 | 4.9 |
| 72 | 600 | 5.3 |
| 72 | 900 | 2.1 |
| 72 | 900 | 8.8 |
| 72 | 1200 | 9.2 |
| 72 | 1200 | 11.6 |
| 72 | 1500 | 8.1 |
| 72 | 1500 | 11.3 |
| 72 | 1500 | 19.7 |
| 72 | 1500 | 13.4 |
| 72 | 1800 | 8.5 |
| 72 | 1800 | 11.6 |
| 72 | 1800 | 18.7 |
| 72 | 2100 | 13.7 |
| 72 | 2100 | 15.2 |
| 72 | 2100 | 20.1 |
| 72 | 2100 | 23.3 |
| 72 | 2100 | 26.1 |
| 72 | 2400 | 24.7 |
| 72 | 2400 | 25.7 |
| 72 | 2400 | 27.1 |
| 72 | 2400 | 31.7 |
| 72 | 2400 | 32.4 |
| 72 | 2400 | 34.5 |
| 72 | 2400 | 35.6 |

Split doses:

| First dose (Gy) | Time after first dose (h) | Second dose (Gy) | Time after second dose (h) | Estimated DSB/cell |
| --- | --- | --- | --- | --- |
| 2400 | 48 | 1200 | 0 | 108.3 |
| 2400 | 48 | 1200 | 24 | 48.8 |
| 2400 | 48 | 1200 | 24 | 51.7 |
| 2400 | 48 | 1200 | 24 | 54.7 |
| 1500 | 24 | 1500 | 0 | 119.1 |
| 1500 | 24 | 1500 | 24 | 36.9 |
| 900 | 16 | 900 | 0 | 70.6 |
| 900 | 16 | 900 | 16 | 10.9 |
| 900 | 16 | 900 | 16 | 23.1 |
| 900 | 16 | 900 | 16 | 35.3 |

(2) Single doses (1250–2400 Gy) of sparsely-ionizing ^60^Co γ-rays delivered at a low dose rate (approximately 33 Gy/h) followed by rejoining time of 0–36 h [[39](#_ENREF_39)]. These data were taken from Figures 1-2 of reference [[39](#_ENREF_39)]. The LET was approximately 0.24 keV/μm.

| Dose (Gy) | Dose rate (Gy/h) | Rejoining time (h) | Estimated DSB/cell |
| --- | --- | --- | --- |
| 1250 | 32.89 | 0 | 8.4 |
| 1250 | 32.89 | 0 | 9.3 |
| 1250 | 32.89 | 34 | 1.2 |
| 1250 | 32.89 | 34 | 3.5 |
| 1650 | 33.00 | 0 | 8.3 |
| 1650 | 33.00 | 0 | 7.8 |
| 1650 | 33.00 | 0 | 8.1 |
| 1650 | 33.00 | 0 | 8.5 |
| 1650 | 33.00 | 0 | 9.0 |
| 1650 | 33.00 | 0 | 10.3 |
| 1650 | 33.00 | 0 | 10.6 |
| 1650 | 33.00 | 22 | 4.2 |
| 1650 | 33.00 | 22 | 5.6 |
| 1650 | 33.00 | 22 | 6.0 |
| 1650 | 33.00 | 22 | 6.4 |
| 1860 | 33.21 | 16 | 5.8 |
| 1860 | 33.21 | 16 | 6.2 |
| 1860 | 33.21 | 16 | 6.6 |
| 1860 | 33.21 | 16 | 8.0 |
| 2400 | 33.00 | 0 | 8.1 |
| 2400 | 33.00 | 0 | 8.1 |
| 2400 | 33.00 | 0 | 8.3 |
| 2400 | 33.00 | 0 | 10.6 |
| 2400 | 33.00 | 0 | 10.9 |
| 2400 | 33.00 | 0 | 11.7 |
| 2400 | 33.00 | 0 | 12.5 |
| 2400 | 33.00 | 0 | 13.6 |
| 2400 | 33.00 | 0 | 14.9 |
| 2400 | 33.00 | 0 | 16.5 |

(3) Single doses (100–600 Gy) of densely-ionizing 3.5 MeV α-particles delivered at a high dose rate (1400 Gy/h) followed by rejoining time of 0–72 h [[40](#_ENREF_40), [41](#_ENREF_41)]. These data were taken from Figure 1 of reference [[40](#_ENREF_40)] and Figure 2 of reference [[41](#_ENREF_41)]. The LET was approximately 113 keV/μm.

| Rejoining time (h) | Dose (Gy) | Estimated DSB/cell |
| --- | --- | --- |
| 0 | 100 | 10.3 |
| 0 | 100 | 14.2 |
| 0 | 100 | 15.1 |
| 0 | 100 | 17.2 |
| 0 | 100 | 19.1 |
| 0 | 180 | 18.7 |
| 0 | 180 | 22.6 |
| 0 | 180 | 23.2 |
| 0 | 180 | 26.8 |
| 0 | 180 | 29.7 |
| 0 | 270 | 35.2 |
| 0 | 270 | 38.4 |
| 0 | 270 | 38.8 |
| 0 | 270 | 48.3 |
| 0 | 360 | 45.6 |
| 0 | 360 | 46.0 |
| 0 | 360 | 46.6 |
| 0 | 360 | 47.0 |
| 0 | 360 | 49.8 |
| 0 | 360 | 52.2 |
| 0 | 200 | 29.8 |
| 0 | 300 | 44.3 |
| 0 | 400 | 59.3 |
| 0 | 600 | 88.4 |
| 1 | 200 | 28.0 |
| 1 | 300 | 41.1 |
| 1 | 400 | 54.9 |
| 1 | 600 | 83.1 |
| 3 | 200 | 27.1 |
| 3 | 300 | 39.8 |
| 3 | 400 | 53.3 |
| 3 | 600 | 79.4 |
| 5 | 200 | 24.0 |
| 5 | 200 | 22.3 |
| 5 | 200 | 25.9 |
| 5 | 300 | 34.7 |
| 5 | 300 | 32.2 |
| 5 | 300 | 37.4 |
| 5 | 400 | 47.1 |
| 5 | 400 | 43.7 |
| 5 | 400 | 50.8 |
| 5 | 600 | 70.2 |
| 5 | 600 | 65.1 |
| 5 | 600 | 75.7 |
| 12 | 200 | 19.4 |
| 12 | 200 | 18.0 |
| 12 | 200 | 20.9 |
| 12 | 300 | 28.9 |
| 12 | 300 | 26.8 |
| 12 | 300 | 31.1 |
| 12 | 400 | 39.2 |
| 12 | 400 | 36.4 |
| 12 | 400 | 42.3 |
| 12 | 600 | 58.4 |
| 12 | 600 | 52.2 |
| 12 | 600 | 63.0 |
| 24 | 200 | 6.8 |
| 24 | 200 | 8.5 |
| 24 | 200 | 10.6 |
| 24 | 300 | 10.6 |
| 24 | 300 | 14.3 |
| 24 | 300 | 18.5 |
| 24 | 400 | 17.5 |
| 24 | 400 | 21.6 |
| 24 | 400 | 25.5 |
| 24 | 600 | 31.4 |
| 24 | 600 | 38.6 |
| 24 | 600 | 45.7 |
| 48 | 200 | 1.8 |
| 48 | 200 | 3.6 |
| 48 | 200 | 5.8 |
| 48 | 300 | 3.4 |
| 48 | 300 | 6.2 |
| 48 | 300 | 9.4 |
| 48 | 400 | 5.2 |
| 48 | 400 | 9.6 |
| 48 | 400 | 14.2 |
| 48 | 600 | 9.4 |
| 48 | 600 | 17.1 |
| 48 | 600 | 26.4 |
| 72 | 200 | 1.5 |
| 72 | 200 | 3.9 |
| 72 | 200 | 6.8 |
| 72 | 300 | 3.1 |
| 72 | 300 | 6.8 |
| 72 | 300 | 11.1 |
| 72 | 400 | 4.9 |
| 72 | 400 | 10.7 |
| 72 | 400 | 17.0 |
| 72 | 600 | 9.6 |
| 72 | 600 | 19.7 |
| 72 | 600 | 30.9 |
