# Supplementary material for: Mechanistic Modeling of Dose and Dose Rate Dependences of Radiation-Induced DNA Double Strand Break Rejoining Kinetics in Saccharomyces cerevisiae
Source: PLoS One. 2016 Jan 7;11(1):e0146407. doi: 10.1371/journal.pone.0146407 (PMC4711806; doi:10.1371/journal.pone.0146407)
Supplement: S2 Appendix — (DOCX) [file pone.0146407.s002.docx]

Analytic solution for the RD model

RD model predictions for the three DSB classes at time T after radiation dose *D* delivered with dose rate R are given by the following equations:

DSB_1_(*D*,T) = –*k*_1_×R×(exp(– (R×*q*_1_+*v*_1_)×*D*/R) – 1)×exp(–*v*_1_×T)/(R×*q*_1_+*v*_1_);

DSB_2_(*D*,T) =(R×*k*_1_×*q*_1_×(R×*q*_2_+*v*_2_)×exp(– ((*q*_1_–*q*_2_)×R+*v*_1_–*v*_2_)×*D*/R)+((*q*_1_–*q*_2_)×R+*v*_1_–*v*_2_)

×(*q*_1_×(*k*_1_+*k*_2_)×R+*k*_2_×*v*_1_)×exp((R×*q*_2_+*v*_2_)×*D*/R) – ((*q*_1_×(*k*_1_+*k*_2_) –*k*_2_×*q*_2_)×R

+*k*_2_×(*v*_1_-*v*_2_))×(R×*q*_1_+*v*_1_))×exp(– (R×*q*_2_+*v*_2_)×*D*/R)×R×exp(–*v*_2_×T)/((R×*q*_2_+*v*_2_)

×((*q*_1_–*q*_2_)×R+*v*_1_–*v*_2_)×(R×*q*_1_+*v*_1_));

DSB_3_(*D*,T) =(R^2^×(R×*q*_1_+*v*_1_)^2^×*q*_2_×((*q*_1_×(*k*_1_+*k*_2_) –*k*_2_×*q*_2_)×R+*k*_2_×(*v*_1_–*v*_2_))

×exp(– (R×*q*_2_+*v*_2_)×*D*/R) –*k*_1_×R^3^×(R×*q*_2_+*v*_2_)^2^×*q*_1_×*q*_2_×exp(– (R×*q*_1_+*v*_1_)×*D*/R)

+((*q*_1_–*q*_2_)×R+*v*_1_–*v*_2_)×(*q*_1_×((*D*×(*k*_1_+*k*_2_+*k*_3_)×*q*_1_–*k*_1_)×*q*_2_–*q*_1_×(*k*_1_+*k*_2_))×*q*_2_×R^4^

+(*D*×*v*_1_×(*k*_1_+2×*k*_2_+2×*k*_3_)×*q*_2_+*D*×*v*_2_×(*k*_1_+*k*_2_+2×*k*_3_)×*q*_1_+(–*k*_1_–2×*k*_2_)×*v*_1_

–*k*_1_×*v*_2_)×*q*_1_×*q*_2_×R^3^+(*D*×*v*_1_^2^×(*k*_2_+*k*_3_)×*q*_2_^2^+(*D*×*v*_2_×(*k*_1_+2×*k*_2_+4×*k*_3_)×*q*_1_

-*k*_2_×*v*_1_)×*v*_1_×*q*_2_+*D*×*k*_3_×*q*_1_^2^×*v*_2_^2^)×R^2^+*v*_1_×*D*×(*v*_1_×(*k*_2_+2×*k*_3_)×*q*_2_+2×*k*_3_×*q*_1_×*v*_2_)

×*v*_2_×R+*D*×*k*_3_×*v*_1_^2^×*v*_2_^2^))/(((*q*_1_–*q*_2_)×R+*v*_1_–*v*_2_)×(R×*q*_1_+*v*_1_)^2^×(R×*q*_2_+*v*_2_)^2^) (A1)

Linear-quadratic (LQ) approximation for the RD model

For the RD model, LQ approximation for the total DSB yield (from all three classes combined) after a relatively low dose *D*, delivered with dose rate R and followed by rejoining time T, is given by Taylor series expansion in the vicinity of *D* = 0:

RD_LQ_ = (*k*_1_×exp(–*v*_1_×T)+exp(–*v*_2_×T)×*k*_2_+*k*_3_)×*D*+(½)×(((*k*_1_×*q*_1_–*k*_2_×*q*_2_)×R–*k*_2_×*v*_2_)×exp(–*v*_2_×T) –*k*_1_×(R×*q*_1_+*v*_1_)×exp(–*v*_1_×T)+R×*k*_2_×*q*_2_)/R×*D*^2^ (A2)

At high dose rate and infinite rejoining time Eq. A2 simplifies to:

RD_LQHDR_ = *k*_3_×*D* + (½)×*k*_2_×*q*_2_×*D*^2^  (A3)

Alternatively, at low dose rate and infinite rejoining time, Eq. A2 simplifies to just:

RD_LQLDR_ = *k*_3_×*D* (A4)

Customized optimization procedure used for fitting the TLK model to data

Due to the presence of nonlinear terms for interactions between DSBs (parameters η_1_, η_2_, and η_1,2_ in Eq. 2 of the main text), the TLK model cannot be solved analytically, and numerical solutions are computationally intensive. To generate best-fit values for model parameters using a practical amount of computational resources, we developed the following customized procedure (in Maple 17® software) to minimize the sum of squared deviations (SS) of model predictions from the data. Under the assumption of Gaussian errors, SS minimization results in log likelihood maximization (Eq. 3 of the main text).

The optimization method was based on the response surface methodology (RSM) [[69](#_ENREF_69)]. When the evaluation of the objective function (SS in this case) is computationally costly, the iterative RSM consists of using information accumulated up to the current iteration (i.e. the values of SS obtained so far) to construct an approximate surface of the SS landscape. The following steps were involved in implementing this approach:

1. Identify plausible initial values for all K_M_ model parameters (ten for the TLK model).
2. Throw random numbers around these initial values (using a log-normal distribution with the mean for each parameter set to the parameter’s initial value from step 1, and a standard deviation of ½ on the log scale) to generate random parameter values.
3. For each set of random parameter values from step 2, solve the TLK model numerically and find the SS value for the analyzed data.
4. Repeat steps 2-3 2×K_M_+1 times and in each case record the parameter value combinations and corresponding SS values.
5. Convert the results from step 4 into a system of 2×K_M_+1 quadratic equations which link model parameter values to SS values by 2×K_M_+1 coefficients.
6. Solve the system of equations from step 5 to find the coefficients. This provides a second-order polynomial approximation for the SS surface (i.e. for its dependence of model parameter values).
7. Find the minimum of the approximate surface from step 6 to estimate the location in parameter space of the optimum model parameter values.
8. Replace the initial parameter values from step 1 with the estimates from step 7.
9. Repeat steps 2-3 one time to generate a new random parameter combination and a new SS value.
10. Out of the accumulated set of 2×K_M_+2 parameter combinations and corresponding SS values, eliminate the parameter combination with the highest (worst) SS value.
11. Repeat steps 5-10 multiple (1000) times, reducing the standard deviation of the log-normal distribution in proportion to the square root of the number of iterations.
12. Repeat steps 1-11 multiple (100) times using different random initial parameter values to increase the probability of finding the global (rather than local) SS minimum.

In this approach, the approximate second-order polynomial SS surface was used to guide the optimization process toward the SS minimum. At each step, the approximate surface was updated to provide increasingly accurate estimates for the location of the SS minimum in parameter space. The use of this approximate surface reduced the number of computationally costly SS evaluations.

The reliability of this method was checked by producing a simplified version of the TLK model with 7 parameters instead of 10 (parameters η_1_, η_2_, and η_1,2_ were set to zero). This simplified version is analytically solvable, and the minimum SS could therefore be found by the SQP algorithm, as well as by the proposed procedure described above. Both methods arrived at the same parameter values, given the same initial conditions.
